# Supplementary material for: Anticipatory smooth pursuit eye movements scale with the probability of visual motion: The role of target speed and acceleration
Source: J Vis. 2025 Jan 3;25(1):2. doi: 10.1167/jov.25.1.2 (PMC11702788; doi:10.1167/jov.25.1.2)
Supplement: Supplement 1 [file jovi-25-1-2_s001.pdf]

Supplementary material

Final models for the LMM analysis

Exp 1A:

$aSPv \sim 1 + P(HS) + (1 + P(HS) \mid participant)$

$aSPv \sim 1 + P(HS)*Tv_{N-1} + (1 + P(HS) + Tv_{N-1} \mid participant)$

Exp 1B, constant speed probability-mixtures:

$aSPv \sim 1 + P(v33) + axis + (1 + P(v33) + axis \mid participant)$

$aSPv \sim 1 + P(v33)*Tv_{N-1} + (1 + P(v33) + Tv_{N-1} \mid participant)$

Exp 2A-B, accelerating target probability-mixtures:

$aSPv \sim 1 + prob + axis + exp + axis{:}exp + (1 + prob + axis \mid participant)$

Exp 3, comparison between fully predictable blocks:

$aSPv \sim 1 + v0*accel + (1 + v0 \mid participant)$

Exp3, categorical model for pairwise comparisons:

$aSpv \sim 1 + condition + (1 \mid participant)$

LMM analysis - result tables

Exp1A: Anticipatory Parameters

	Dependent variable:
	aSPv
P(HS)	3.488*** (3.138, 3.838)
	t = 19.541
	p = 0.000
Constant	2.759*** (2.036, 3.482)
	t = 7.480
	p = 0.000
Random Effects	
Groups	3
sd(Constant)	0.77
sd(P(HS))	0.33
Note:	*p < 0.01; **p < 0.001; ***p < 1e-04

Exp 1A: Anticipatory Parameters– Sequential Effects

	Dependent variable:
	aSPv
Tv_{N-1}[LS]	-0.501^{***} (-0.663, -0.340)
	t = -6.073
	p = 0.000
P(HS)	4.357^{***} (3.983, 4.732)
	t = 22.827
	p = 0.000
P(HS):Tv_{N-1}[LS]	-2.744^{***} (-3.102, -2.386)
	t = -15.030
	p = 0.000
Constant	4.427^{***} (3.801, 5.053)
	t = 13.853
	p = 0.000
Random Effects	
Groups	3
sd(Constant)	0.70
sd(P(HS))	0.10
sd(Tv_{N-1}[LS])	0.29
Note:	$^{*}p<0.01$; $^{**}p<0.001$; $^{***}p<1e-04$

Exp 1B: Anticipatory Parameters – Const Speed

	Dependent variable:
	aSPv
P(v33)	2.743*** (1.824, 3.663)
	t = 5.847
	p = 0.000
Axis[vert.]	-0.740 (-1.464, -0.016)
	t = -2.002
	p = 0.046
P(v33):Axis[vert.]	-0.674*** (-0.985, -0.363)
	t = -4.252
	p = 0.00003
Constant	2.707*** (1.740, 3.673)
	t = 5.490
	p = 0.00000
Random Effects	
Groups	13
sd(Constant)	1.82
sd(P(v33))	1.68
sd(Axis[vert.])	1.32
Note:	*$p < 0.01$; **$p < 0.001$; ***$p < 1e\text{-}04$

Exp 1B: Anticipatory Parameters – Const Speed - Sequential Effects

	Dependent variable:
	aSPv
Tv_{N-1}[v11]	-0.613*** (-0.882, -0.345)
	t = -4.482
	p = 0.00001
P(v33)	1.380* (0.437, 2.323)
	t = 2.869
	p = 0.005
Tv_{N-1}[v11]:P(v33)	0.823** (0.383, 1.263)
	t = 3.663
	p = 0.0003
Constant	3.948*** (3.114, 4.781)
	t = 9.279
	p = 0.000
Random Effects	
Groups	13
sd(Constant)	1.58
sd(P(v33))	1.67
sd(Tv_{N-1}[v11])	0.43
Note:	*p<0.01; **p<0.001; ***p<1e-04

Exp 2A-B: Anticipatory Parameters – Accelerating Target

	Dependent variable:
	aSPv
P(vdec)	1.884^{***} (1.111, 2.657)
	t = 4.779
	p = 0.00001
Axis[vert.]	-0.460 (-1.264, 0.343)
	t = -1.123
	p = 0.262
Exp.[const.Time]	1.683^{***} (1.410, 1.956)
	t = 12.086
	p = 0.000
Exp.[const.Time]:Axis[vert.]	-1.651^{***} (-2.027, -1.275)
	t = -8.611
	p = 0.000
Constant	3.041^{***} (2.099, 3.983)
	t = 6.326
	p = 0.000
Random Effects	
Groups	16
sd(Constant)	1.96
sd(P(V3))	1.58
sd(Axis[vert.])	1.66
Note:	$^{*}p<0.01$; $^{**}p<0.001$; $^{***}p<1e\text{-}04$

Exp 3: Anticipatory Parameters – Parametric Variables

	Dependent variable:
	aSPv
V0	0.246*** (0.149, 0.343)
	t = 4.963
	p = 0.00000
Accel	0.017* (0.004, 0.030)
	t = 2.640
	p = 0.009
V0:Accel	0.001* (0.0004, 0.002)
	t = 2.905
	p = 0.004
Constant	0.364 (-0.324, 1.052)
	t = 1.037
	p = 0.300
Random Effects	
Groups	7
sd(Constant)	0.95
sd(v0)	0.14
Groups	7
Note:	*p < 0.01; **p < 0.001; ***p < 1e-04

Exp 3: Pairwise comparisons

contrast	estimate	SE	df	t.ratio	p.value
V1a - V1c	0.640	0.140	8749	4.559	<.0001
V1a - V1d	0.934	0.143	8749	6.545	<.0001
V1a - V2a	-2.236	0.136	8749	-16.396	<.0001
V1a - V2c	-1.037	0.140	8749	-7.430	<.0001
V1a - V2d	-1.503	0.139	8749	-10.779	<.0001
V1a - V3a	-4.114	0.130	8749	-31.598	<.0001
V1a - V3c	-3.320	0.135	8749	-24.626	<.0001
V1a - V3d	-2.679	0.136	8749	-19.647	<.0001
V1c - V1d	0.294	0.142	8749	2.065	0.0390
V1c - V2a	-2.876	0.136	8749	-21.149	<.0001
V1c - V2c	-1.677	0.139	8749	-12.051	<.0001
V1c - V2d	-2.143	0.139	8749	-15.417	<.0001
V1c - V3a	-4.754	0.130	8749	-36.624	<.0001
V1c - V3c	-3.960	0.134	8749	-29.472	<.0001
V1c - V3d	-3.319	0.136	8749	-24.415	<.0001
V1d - V2a	-3.170	0.138	8749	-22.921	<.0001
V1d - V2c	-1.971	0.141	8749	-13.932	<.0001
V1d - V2d	-2.437	0.141	8749	-17.251	<.0001
V1d - V3a	-5.048	0.132	8749	-38.177	<.0001
V1d - V3c	-4.254	0.137	8749	-31.095	<.0001
V1d - V3d	-3.613	0.138	8749	-26.115	<.0001
V2a - V2c	1.199	0.135	8749	8.875	<.0001
V2a - V2d	0.733	0.135	8749	5.434	<.0001
V2a - V3a	-1.878	0.125	8749	-14.982	<.0001

V2a - V3c	-1.084	0.130	8749	-8.326	<.0001
V2a - V3d	-0.443	0.132	8749	-3.362	0.0008
V2c - V2d	-0.466	0.138	8749	-3.372	0.0008
V2c - V3a	-3.077	0.129	8749	-23.872	<.0001
V2c - V3c	-2.283	0.134	8749	-17.100	<.0001
V2c - V3d	-1.642	0.135	8749	-12.153	<.0001
V2d - V3a	-2.611	0.129	8749	-20.288	<.0001
V2d - V3c	-1.818	0.133	8749	-13.631	<.0001
V2d - V3d	-1.176	0.135	8749	-8.719	<.0001
V3a - V3c	0.794	0.124	8749	6.413	<.0001
V3a - V3d	1.435	0.125	8749	11.442	<.0001
V3c - V3d	0.641	0.130	8749	4.927	<.0001

Degrees-of-freedom method: containment

P value adjustment: BH method for 36 tests
